# Anal extrusion of a ventriculoperitoneal shunt in a 1-month-old patient: a case report

**DOI:** 10.1093/jscr/rjae306

**Published:** 2024-05-14

**Authors:** I Gusti Ketut Agung Surya Kencana, Sri Maliawan, Christopher Lauren, Anak Agung Ngurah Agung Harawikrama Adityawarma, Denny Japardi

**Affiliations:** Neurosurgery Division, Department of Surgery, Faculty of Medicine, Universitas Udayana, Prof. Dr. I.G.N.G. Ngoerah General Hospital, Denpasar, Bali, Indonesia; Neurosurgery Division, Department of Surgery, Faculty of Medicine, Universitas Udayana, Prof. Dr. I.G.N.G. Ngoerah General Hospital, Denpasar, Bali, Indonesia; Neurosurgery Division, Department of Surgery, Faculty of Medicine, Universitas Udayana, Prof. Dr. I.G.N.G. Ngoerah General Hospital, Denpasar, Bali, Indonesia; Neurosurgery Division, Department of Surgery, Faculty of Medicine, Universitas Udayana, Prof. Dr. I.G.N.G. Ngoerah General Hospital, Denpasar, Bali, Indonesia; Neurosurgery Division, Department of Surgery, Faculty of Medicine, Universitas Udayana, Prof. Dr. I.G.N.G. Ngoerah General Hospital, Denpasar, Bali, Indonesia

**Keywords:** hydrocephalus, intestinal perforation, neurosurgery, pediatric, ventriculoperitoneal shunt

## Abstract

The ventriculoperitoneal shunt procedure represents a frequently conducted neurosurgical intervention; nevertheless, it harbors inherent risks that can precipitate complications in patients. Intestinal perforation accompanied by distal shunt protrusion through the anus is an uncommon phenomenon, observed in ~0.1% to 0.7% of cases, with mortality rates reaching up to 15%. Timely identification and comprehensive management of such complications are imperative to prevent further deterioration of the patient’s condition. Herein, we present a case involving a 1-month-old female infant who presented with a tube protruding from the anal orifice. Immediate surgical intervention was undertaken to remove the distal shunt and prevent further infection in the patient.

## Introduction

The ventriculoperitoneal (VP) shunt procedure is a commonly conducted neurosurgical technique designed to redirect cerebrospinal fluid from the ventricles to the abdominal cavity. Despite its widespread use, this procedure carries inherent risks that may result in complications for patients. Studies have indicated that abdominal complications following VP shunt procedures occur in up to 25% of cases. Among these, instances of intestinal perforation accompanied by distal shunt protrusion through the anus are rare, occurring in ~0.1% to 0.7% of cases, with mortality rates reaching up to 15% of cases [1–3].

In this report, we present an intriguing case involving a patient who experienced distal shunt migration, resulting in protrusion through the anus. We provide a detailed account of this case, drawing upon relevant literature and previously documented case studies.

## Case report

**Figure 1:**
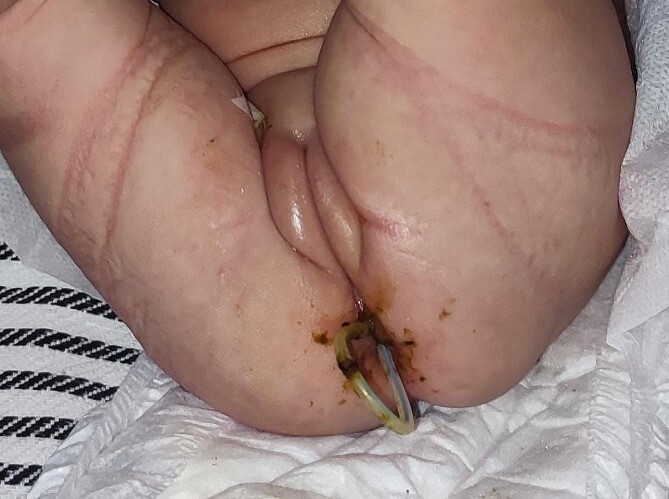
The appearance of a tube shunt emerging from the anal orifice.

**Figure 2:**
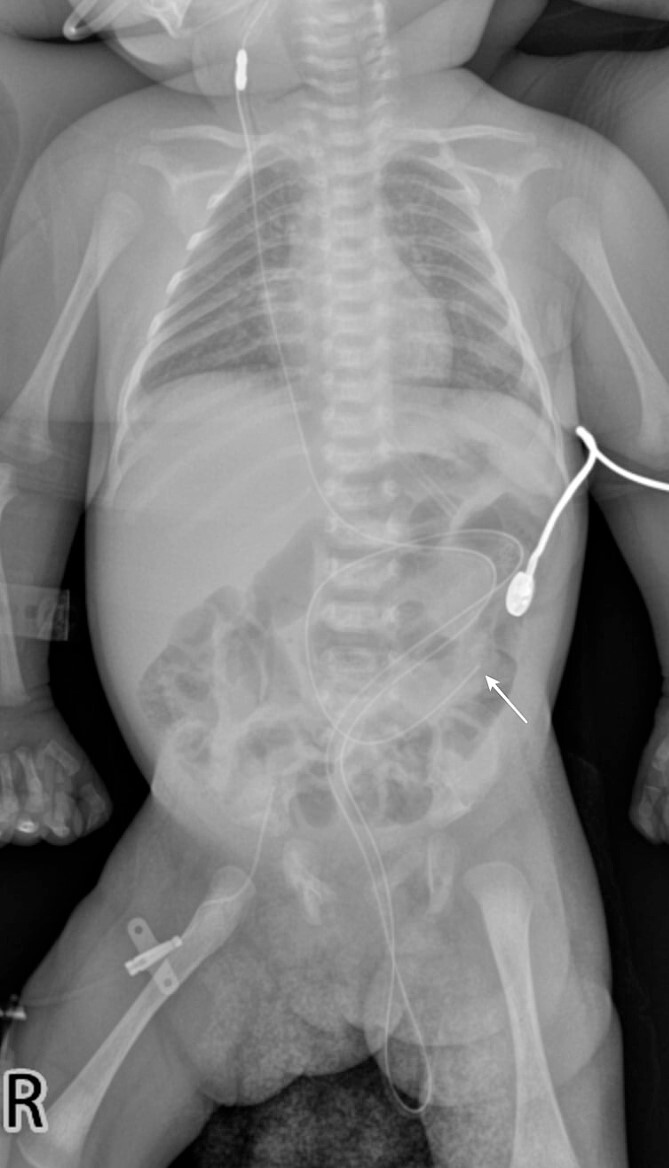
A babygram X-ray demonstrates the distal tip of the shunt positioned at the level of the left paravertebral L4 (white arrow), with a portion of the shunt extending downward and protruding from the abdominal cavity.

A 1-month-old female infant presented with a protruding tube from the anal orifice, 1 month following VP shunt placement for congenital hydrocephalus. She displayed no signs of shunt malfunction or gastrointestinal disturbances. Examination confirmed the presence of the distal VP shunt tube protruding from the anus (Fig. 1), as verified by babygram X-ray (Fig. 2). Immediate laparotomy was undertaken to remove the shunt. Intraoperatively, it was revealed that the shunt had breached a perforation in the descending colon. The proximal shunt was disconnected and removed, while the distal segment was retracted into the abdomen and gently guided through the anus, followed by meticulous abdominal closure. The decision was made not to close the colonic perforation, as the surrounding fibrous tissue was deemed sufficient to contain abdominal contents. Postoperatively, the patient underwent a 1-day fasting period before a gradual dietary reintroduction. Discharge ensued after 7 days, with prophylactic antibiotics administered. Subsequent follow-up appointments demonstrated no complications, with the patient maintaining shunt independence and exhibiting good health.

## Discussion

Although VP shunt is frequently performed, it is not without complications that may occur in patients. The risk of abdominal complications due to VP shunt procedures is reported to reach an incidence rate of 25% of the total number of cases. Of this number, the incidence of cases with intestinal perforation accompanied by distal shunt protrusion through the anus orifice reaches a total of 0.1%–0.7% of cases [1, 2]. Intestinal perforation is a rarely encountered complication of VP shunt placement, yet it can lead to serious complications for patients, with mortality reaching 15% of cases [3].

Most patients do not show symptoms or are asymptomatic in the majority of reported cases [4]. Several mechanisms causing perforation have been proposed by many experts. These factors include foreign body reactions, pressure necrosis on the intestinal wall due to pressure from the tube, and allergy to the silicone material of the shunt [4, 5]. Contributing factors include the thin and lax musculature of the infant intestine, making perforation more likely [6]. Some experts recommend anchoring the distal end of the shunt to the peritoneum [7]. However, in our case, the shunt tip remained within the peritoneal cavity, and what entered the perforation hole was part of the distal shunt. Perforation of the intestinal lumen can occur due to the catheter tube moving freely within the peritoneal cavity and pressing on the thin serosal layer of the infant intestine, leading to necrosis and perforation in that area. Subsequently, intestinal peristalsis causes the shunt tube to continue moving forward until it exits through the anus orifice [8].

Management of patients with this condition includes operative procedures to remove the shunt, infection control, and timely reinsertion of the shunt. In some asymptomatic cases, removal of the shunt via endoscopy or colonoscopy through the anus can be performed after disconnecting from the tube through an incision in the neck [4, 5]. The fibrous tissue covering the perforated area typically provides adequate protection, preventing spillage of intestinal contents into the peritoneal cavity [9]. However, in our case, there were no facilities, such as endoscopy, so it was recommended to perform laparotomy through the previous surgical scar from the shunt placement operation. This approach ensures enhanced safety as direct visualization of the shunt and detection of intra-abdominal content spillage into the peritoneal cavity are facilitated. Laparotomy is also required in cases of intra-abdominal infection, such as peritonitis or abscesses, or fistulas that do not close spontaneously during the shunt removal process via endoscopy [9, 10].

Several preventive measures can be taken to prevent shunt migration. The type of catheter most commonly associated with perforation is the Raimondi spring-coiled catheter. The use of softer and more flexible silastic tubing can reduce the incidence of bowel perforation [11]. Additionally, anchoring the distal end of the shunt to the peritoneum can prevent shunt migration in patients [7]. Patients with poor nutritional status also significantly increase the risk of bowel perforation, necessitating the pursuit of their nutritional needs, particularly in infants, to avoid this complication [12].

It is essential to prioritize patient safety and enhance their quality of life in the management of medical conditions, especially in pediatric patients. The following measures can help in managing or mitigating events related to the use of shunt devices in pediatric patients. First, selecting the appropriate shunt device, such as choosing a suitable catheter type, like silicone catheters that are softer and more flexible, can reduce the risk of bowel perforation or shunt migration. Second, handling and implanting the shunt device with caution. The shunt placement procedure should be performed by trained and experienced medical personnel to reduce the risk of complications. This includes ensuring that the distal end of the shunt is properly placed in the peritoneum to prevent migration. Third, monitoring and supervision of patients. It is crucial to regularly monitor patients after shunt placement to promptly detect signs of complications or shunt failure. Fourth, providing education and support to parents. Parents should be provided with adequate information about danger signs and post-operative care so that they can recognize symptoms that need attention and act swiftly if necessary. Finally, nutritional management. Pediatric patients with poor nutritional status have a higher risk of complications, including bowel perforation. Therefore, it is important to ensure that patients receive sufficient nutrition to support their recovery.
